# NEAT1/miR-101-dependent Up-regulation of DNA-PKcs Enhances Malignant Behaviors of Pancreatic Ductal Adenocarcinoma Cells

**DOI:** 10.7150/jca.58824

**Published:** 2021-07-25

**Authors:** Hao Hu, Wuqiang Chen, Shuo Zhang, Yuzheng Xue, Youzhao He, Yuanlong Gu

**Affiliations:** 1 Hepatobiliary and Pancreatic Surgery, Affiliated Hospital of Jiangnan University, 585 Xingyuan Rd, Liangxi District, Wuxi, 214041, China; 2 School of Medicine, Jiangnan University, Wuxi 214122, China; 3 Hepatobiliary and Pancreatic Surgery, The Third Hospital Affiliated to Nantong University, Wuxi 214041, China; 4 Medical School, Nantong University, Nantong 226001, China; 5 Wuxi Institute of Hepatobiliary Surgery, Wuxi 214041, China; 6 Department of Gastroenterology, Affiliated Hospital of Jiangnan University, Wuxi 214041, China

**Keywords:** DNA-PKcs, NEAT1, miR-101, pancreatic ductal adenocarcinoma

## Abstract

**Background:** Although we previously revealed that DNA-dependent protein kinase catalytic subunit (DNA-PKcs) is overexpressed in pancreatic ductal adenocarcinoma (PDAC) and important for gemcitabine resistance, the role of DNA-PKcs in the progression and metastasis of PDAC remain unclear. To date, the upstream signaling events stimulating DNA-PKcs overexpression in PDAC are still not well characterized.

**Methods:** Expression of DNA-PKcs was measured by western blot. The levels of miRNA-101 and lncRNA nuclear paraspeckle assembly transcript 1 (NEAT1) were detected by real-time PCR. Cell viability was determined by CCK-8. Cell migration and cell invasion were measured by transwell assay. The regulatory relationship between NEAT1 and miR-101 was determined by a luciferase assay.

**Results:** DNA-PKcs expression was significantly elevated in human PDAC tissues and cells. DNA-PKcs overexpression was correlated with TNM stage and lymph node metastasis. Higher expression of DNA-PKcs was closely correlated with patients of worse overall survival (OS). DNA-PKcs knockdown suppresses malignant behaviors of PDAC cells. Further study showed that miRNA-101 level was decreased in PDAC tissues and cells, which could be responsible for DNA-PKcs overexpression and DNA-PKcs mediated oncogenic actions in PDAC cells. Moreover, NEAT1 functions as an oncogene influencing cell proliferation, migration and invasion in part by serving as a competing endogenous RNA (ceRNAs) modulating miR-101 expression, leading to up-regulation of DNA-PKcs.

**Conclusion:** These findings suggest that NEAT1/miR-101-dependent up-regulation of DNA-PKcs promotes the malignant behaviors of PDAC cells. The NEAT1/miR-101/DNA-PKcs axis may serve as a viable prognostic marker and therapeutic target for PDAC.

## Introduction

Pancreatic cancer (PC) is one of the deadliest cancers in the world, with a high incidence and mortality rate 1-3. Pancreatic ductal adenocarcinoma (PDAC) accounts for approximately 90% of PC 1-3. Despite the recent improvement in methods of treatments for PDAC**,** the 5-year survival rate for patients is lower than 10% due to high invasiveness and early metastasis 1-3. Therefore, understanding the potential mechanism of PDAC progression is essential to exploring effective treatment methods for this deadly disease.

DNA-dependent protein kinase catalytic subunit (DNA-PKcs), a member of the phosphatidylinositol-3 kinase-like protein kinase (PIKK) family, is best known as a mediator of the cellular response to DNA damage 4. DNA-PKcs also has multiple cellular functions, including the maintenance of telomeres, progression of the cell cycle and the regulation of transcription 5. It has been demonstrated that DNA-PKcs plays an important role in initiation, progression, metastasis and chemo/radio-resistance in different types of cancer 6-9, and thus as an emerging therapeutic target in cancer 10, 11. Our previous studies revealed that DNA-PKcs was up-regulated in PDAC and important for gemcitabine resistance 12, 13. However, little is known about the role of DNA-PKcs in the progression and metastasis of PDAC. To date, the molecular mechanism that modulates DNA-PKcs overexpression in PDAC is still poorly understood.

Recent studies showed that long noncoding RNAs (lncRNAs) regulates the initiation, progression, metastasis and chemo/radio-resistance of PDAC 14-16. LncRNA nuclear paraspeckle assembly transcript 1 (NEAT1) has been reported to be dysregulated in many human cancers and contribute to cancer development 17-21. However, the function and the potential mechanism of NEAT1 in the progression of PDAC remain elusive. A recent study by Feng *et al.* found that NEAT1 promotes growth and metastasis of PC by stabilizing ELF3 mRNA 22. Luo *et al.* showed that NEAT1 promotes PDAC cell migration and proliferation by forming a feedback loop with RELA and miR-302a-3p 23.

It is well known that lncRNAs interact with miRNAs and serve as miRNA “sponges” to inhibit miRNA expression, eventually lead to the up-regulation of target gene 24. NEAT1 was reported to serve as a “sponge” of miR-101 to promote the progression and radio-resistance of some types of cancer, including non-small cell lung cancer, hepatocellular carcinoma, papillary thyroid carcinoma, breast cancer and nasopharyngeal carcinoma 25-29. MiR-101 was found to be an anti-oncogenic gene in PDAC. Furthermore, our and previous studies demonstrated that miR-101 selectively targets and downregulates DNA-PKcs 13, 30. These prompted us to investigate whether NEAT1 regulates DNA-PKcs expression by sponging miR-101 and their effects on the progression of PDAC.

In this study, the expression pattern and functions of DNA-PKcs in PDAC were investigated. Furthermore, the regulatory effects and mechanisms of NEAT1 and miR-101 on DNA-PKcs expression were examined. In summary, our results demonstrated that NEAT1/miR-101-dependent up-regulation of DNA-PKcs promotes PDAC cell proliferation, migration and invasion.

## Materials and Methods

### Tissue samples, cell lines and cell transfection

PC and corresponding adjacent normal tissue samples were collected from 30 patients who underwent pancreatic resection at the Hepatobiliary and Pancreatic Surgery, Affiliated Hospital of Jiangnan University, from Feb 2014 to Sep 2019. The protocol was approved by the Ethics Committee of Affiliated Hospital of Jiangnan University and all patients signed a written informed consent form before specimen collection. All PC and matched non-tumor specimens were diagnosed by pathology. None of the patients received radiotherapy and/or chemotherapy before surgery. The inclusion criteria include (1) histologically confirmed diagnosis and (2) no previous treatment. The exclusion criteria include (1) serious complications, (2) presence of other malignant diseases, or (3) incomplete follow-up data. The resected specimens were immediately frozen by liquid nitrogen until further use. The clinicopathologic characteristics of patients are detailed in Table 1. Follow-up data were collected for all subjects and the overall survival time was calculated from the date of surgery to the date of death or the end of follow-up (Apr 2020).

Human PDAC cell lines (AsPC-1, BxPC-3, MIA PaCa-2 and PANC-1) and normal pancreatic cells (HPDE6C7) were purchased from Shanghai Zhong Qiao Xin Zhou Biotechnology Co., Ltd (Shanghai, China). Cells were cultured in specific medium (all from Shanghai Zhong Qiao Xin Zhou Biotechnology Co., Ltd) in humidified air at 37˚C with 5% CO_2_. Cells were routinely tested for mycoplasma contamination. shRNA scramble control (sh-NC), Short hairpin RNA (shRNA) targeting NEAT1 (sh-NEAT1), miR-101 mimic or miR-101 inhibitor and their corresponding negative control were obtained from Genepharma (Shanghai, China). These oligonucleotides were transfected into PANC-1 and MIA PaCa-2 using Lipofectamine 2000 reagent (Invitrogen, Carlsbad, CA, USA) according to the protocol of the manufacturer.

### Reverse transcription-quantitative polymerase chain reaction (RT-qPCR)

TRIzol reagent (Invitrogen, Carlsbad, USA) was used to extract total RNA from PDAC cells. First-strand cDNA was synthesized using a reverse transcriptase kit (Applied Biosystems, Foster City, CA, USA) according to the manufacturer's instructions. The mirVana qRT-PCR miRNA detection kit (Ambion, Austin, U.S.A.) in conjunction with SYBR Green PCR Kit (Thermo Fisher Scientific, MA, U.S.A.) was used for miRNA quantitative PCR. 2^-ΔΔCt^ method was used to calculate the relative expression and the U6 was used as internal reference.

### Western blot

Proteins were extracted using RIPA lysis buffer containing 1% PMSF and cocktail (Beyotime, Haimen, China). The protein concentrations were measured by BCA assay. The proteins were separated by SDS-PAGE and transferred to polyvinylidene difluoride (PVDF) membranes. After blocking with non-fat milk (5%), the membranes were then incubated overnight at 4 °C with the following antibodies: anti-DNA-PKcs (abcam, ab32566, 1:5000), anti-vimentin (CST, 5741, 1:1000) anti-E-cadherin (bioworld, bs1098, 1: 1000), anti-intergrin-β4 (abcam, ab182120, 1:1000) and anti-GAPDH (CST, 5174, 1:1000). The membranes were washed thrice with TBST buffer and then incubated with secondary antibodies at room temperature for 1 h. Enhanced chemiluminescence (ECL) reagent (Thermo Fisher Scientific, Inc.) was used for the detection of interest protein bands. Image J v1.48u software (National Institutes of Health, Bethesda, MD) was employed to analyze the relative optical densities of interest bands.

### CCK-8 assay

The viability of PDAC cells was measured using CCK-8 (Roche Diagnosis, Mannheim, Germany). The cells were seeded into 96-well plate at a density of 5×10^3^cells/well and cultured for 24h, then the cells were transfected as described in the text. After transfection for 48 h, 20 μL CCK-8 was replenished to each well. The absorbance was appraised using a plate reader (ELx800, BioTek Instruments, Inc., Vermont, USA) at 450 nm after 2 h of incubation.

### Cell migration and invasion assays

Cell migration was determined by the transwell assay. PDAC cells were transfected as described. 1×10^5^ cells in serum-free medium were added to the top chamber. The lower chamber was filled with medium containing 10% FBS as a chemoattractant. Cells on the upper surface of the top chamber were removed with cotton swabs after incubation for 24 h. Migrated cells were fixed with methanol for 30 min and stained with 5% crystal violet for 20 min. The cells from each chamber were counted by Leica microscope (Leica DM2500; Leica Microsystems). The number of migrated cells was counted from five randomly selected fields of view. Cell invasion was measured using Matrigel‑coated (dilution 1:3; cat. no. 354234; BD Biosciences) chambers and the other steps were consistent with cell migration assay.

### Luciferase reporter assay

The wild type (WT) or mutant (MUT) luciferase reporter vector of NEAT1 or DNA-PKcs 3ʹUTR containing with miR-101 binding sequences was synthesized by Promega (Shanghai, China). HEK293T cells were cotransfected with the indicated vectors and miR-101 mimics or miR-101 inhibitor using Lipofectamine 2000 reagent (Invitrogen). The relative luciferase activity was analyzed using a dual-luciferase reporter assay kit (Promega) according to the protocol of the manufacturer.

### Statistical analysis

All quantitative data were presented as mean ± SD. Fisher's exact test or Chi-Square test, if appropriate, were used for categorical variables, and Student's t test or ANOVA for quantitative variables. Differences in patient survival were performed using the Kaplan-Meier method and analyzed by log-rank test. The relative risk for each factor was evaluated using univariate and multivariate Cox regression analysis. Correlation analysis was explored by Pearson's correlation. Statistical analysis and graph presentation were performed using SPSS v.17.0 software (SPSS Inc., Chicago, IL) and GraphPad Prism 5 software (GraphPad, San Diego, CA). A P value of <0.05 was regarded as statistically significant.

## Results

### DNA-PKcs is up-regulated in PDAC tissues and cell lines

**Figure 1:**
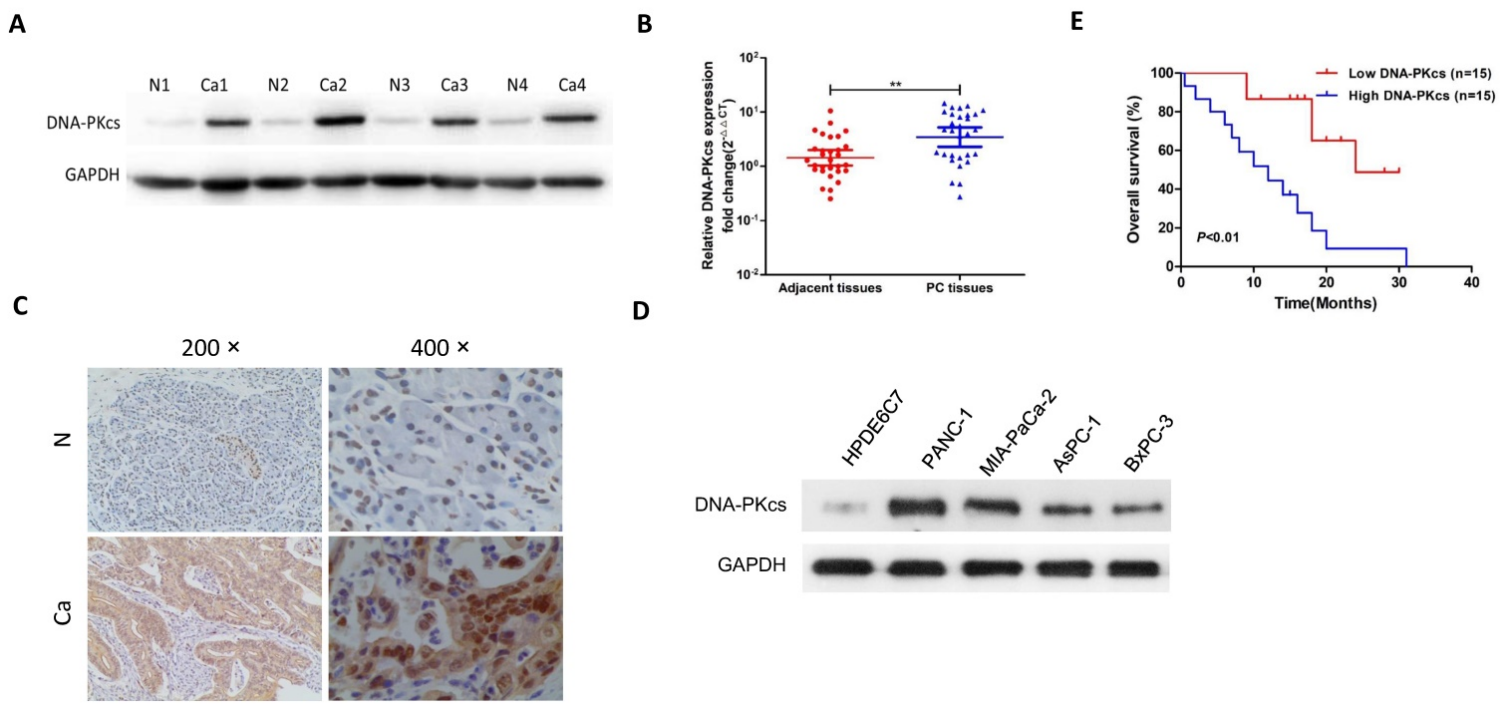
** The expression of DNA-PKcs in human PDAC tissues and survival analysis.** (A) Protein level of DNA-PKcs was determined by western blotting in four independent PDAC tissues. GAPDH was loaded as control. (B) The mRNA level of DNA-PKcs was determined by qRT-PCR in 30 paired PDAC tissues. (C) Representative DNA-PKcs staining of PDAC tissues is shown at 200× and 400× magnifications. (D) Protein level of DNA-PKcs was determined by western blotting in 4 PDAC cell lines (AsPC-1, BxPC-3, MIA PaCa-2 and PANC-1) and normal pancreatic cells (HPDE6C7). (E) Kaplan-Meier survival curves for OS in PDAC patients with different DNA-PKcs protein levels. N, adjacent normal tissues; Ca, PDAC tissues. **P<0.01 compared with adjacent normal tissues.

First, the expression pattern of DNA-PKcs was exaimed in human PDAC tissues, 4 PDAC cell lines and the normal human pancreatic ductal epithelial cell line, HPDE6C7. As shown in Fig 1A, the protein level of DNA-PKcs in PDAC tissues was significantly up-regulated in the examined four independent PDAC tissues. The qRT-PCR result showed that the DNA-PKcs mRNA level was also up-regulated in PDAC tissues (Fig. 1B). The expression of DNA-PKcs was further analyzed using IHC in 30 paired PDAC tissues. As shown in Fig 1C, the PDAC tissues exhibited strong immunoreactivity for DNA-PKcs. DNA-PKcs expression was also higher in 4 PDAC cell lines (AsPC-1, BxPC-3, MIA PaCa-2 and PANC-1) than normal pancreatic cells (HPDE6C7, Fig. 1D).

The correlation of DNA-PKcs and various clinicopathological features was then investigated. As shown in Table 1, DNA-PKcs expression was not associated with age, gender and T stage of PDAC. DNA-PKcs high-expression was positively associated with TNM stage and lymph node metastasis. Kaplan-Meier survival analysis revealed that patients with DNA-PKcs high-expression PDAC had an obviously worse overall survival (OS) than that of low-expression (Fig. 1E).

### DNA-PKcs knockdown suppresses malignant behaviors of PDAC cells

**Figure 2:**
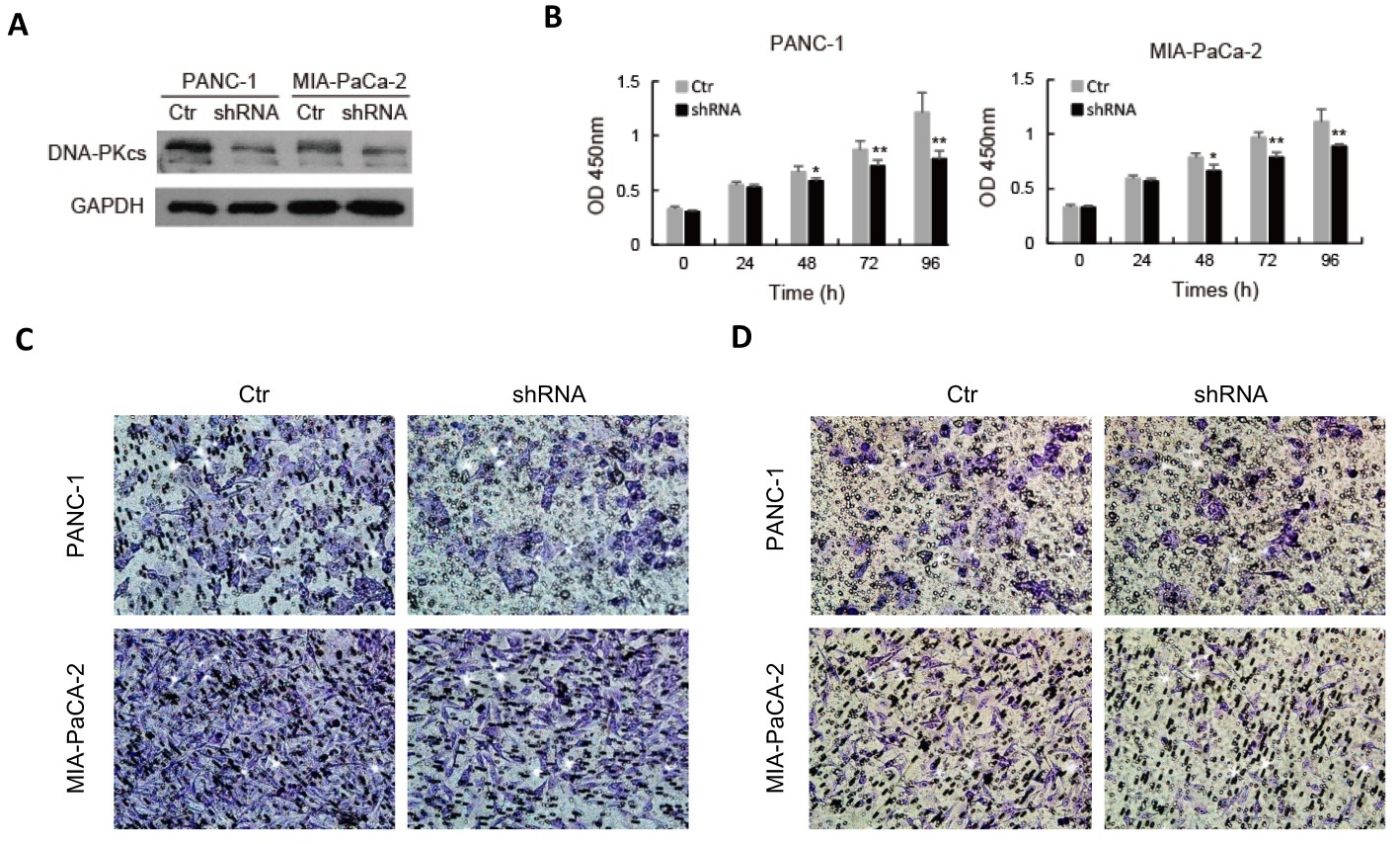

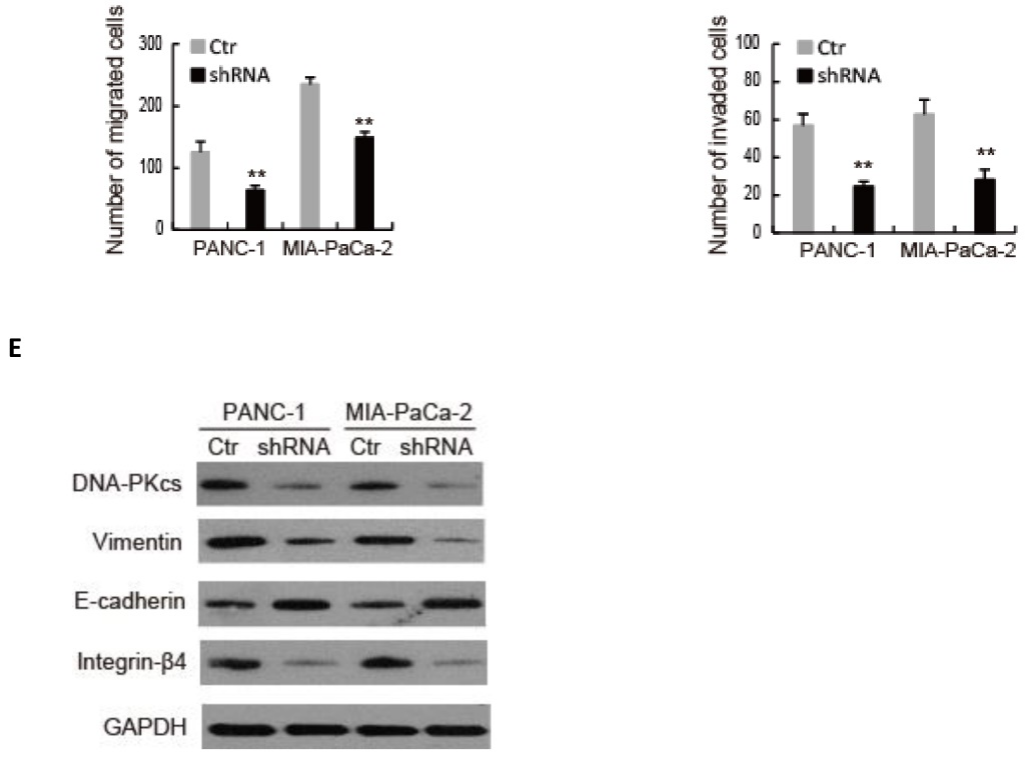
** DNA-PKcs silence suppresses cell proliferation, migration, invasion and EMT in PDAC cells.** PANC-1 and MIA PaCa-2 cells were transfected with DNA-PKcs shRNA (shRNA) or control shRNA (Ctr). (A) The level of DNA-PKcs was examined by western blot. (B) Cell viability was analyzed by CCK-8 assay. (C-D) The numbers of migration and invasion cells were detected by transwell assay. (E) The levels of EMT markers were examined by western blot. *P<0.05, **P<0.01compared with Ctr.

To explore the role of DNA-PKcs in PDAC progression, endogenous DNA-PKcs expression was inhibited by inserting shRNA into PANC-1 and MIA PaCa-2 cells, which showed a high expression level of DNA-PKcs (Fig. 1D). Western blot confirmed that DNA-PKcs‑specific shRNA significantly suppressed DNA-PKcs expression (Fig. 2A). The CCK-8 assay demonstrated that DNA-PKcs shRNA inhibited proliferation of PANC-1 and MIA PaCa-2 cells (Fig. 2B). The migration and invasion assays showed that DNA-PKcs knockdown resulted in significant inhibitions of migration and invasion in PANC-1 and MIA PaCa-2 cells (Fig. 2C and 2D). DNA-PKcs knockdown also led to the inhibition of EMT, with decreased levels of vimentin, integrin-β4 and increased level of E-cadherin (Fig. 2E). These data suggested that DNA-PKcs serves as a tumor promoter by promoting cell proliferation, migration and invasion in PDAC.

### DNA-PKcs serves as a direct target of miR-101 to regulate the malignant behaviors of PDAC cells

**Figure 3:**
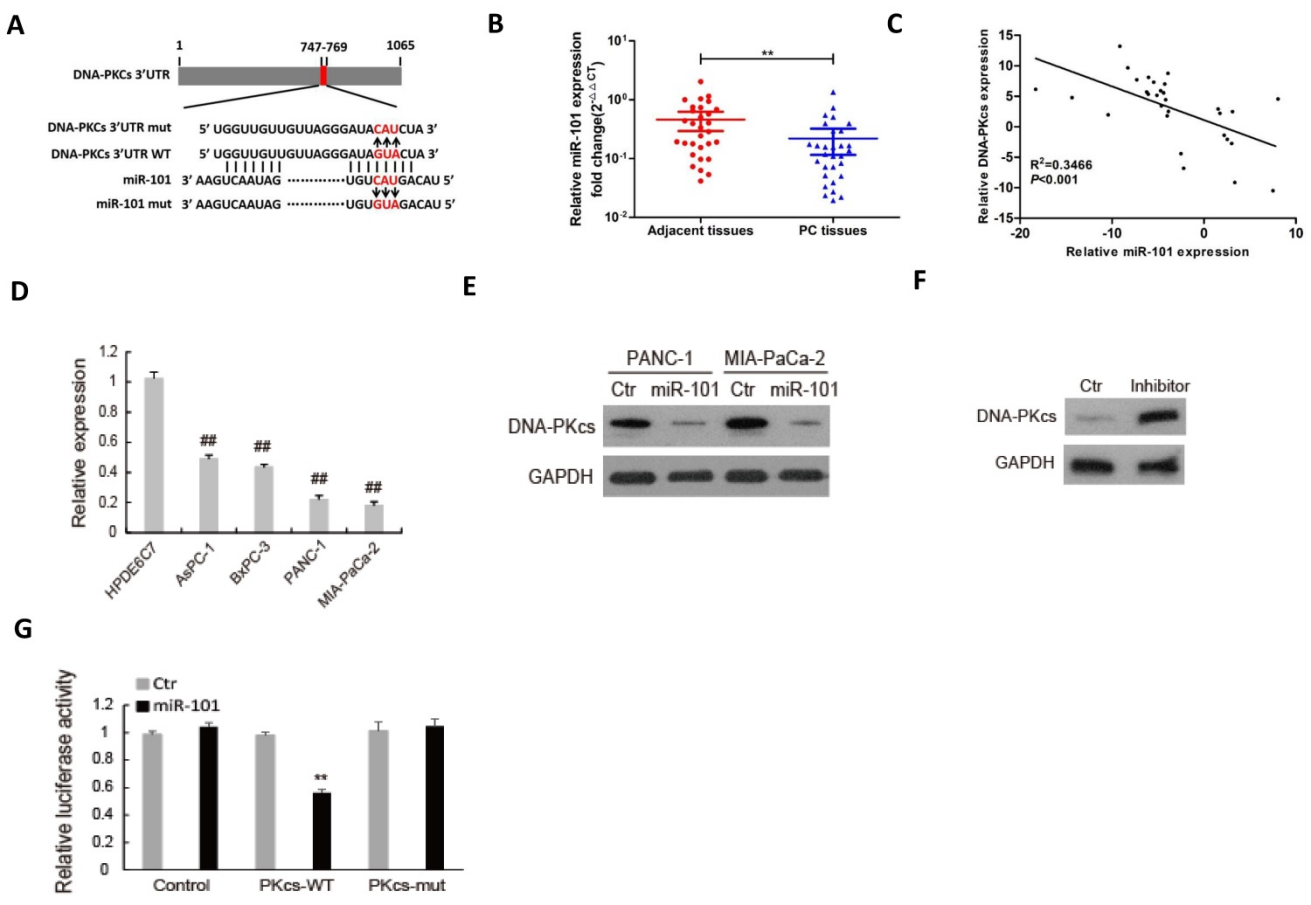
** DNA-PKcs is a direct target of miR-101 in PDAC cells.** (A) The binding condition between miR-101 and DNA-PKcs were predicted, and the putative binding sites was indicated by red bases. (B) The level of miR-101 was determined by qRT-PCR in 30 paired PDAC tissues. (C) The correlation analysis of miR-101 and DNA-PKcs expression. (D) The expression level of miR-101 was determined by qPCR in 4 PDAC cell lines (AsPC-1, BxPC-3, MIA PaCa-2 and PANC-1) and normal pancreatic cells (HPDE6C7). (E) The expression of DNA-PKcs in PANC-1 and MIA PaCa-2 cells transfected with miRNA negative control (Ctr) or miR-101 mimic (miR-101) was measured by using western blot. (F) The expression of DNA-PKcs in HPDE6C7 cells transfected with inhibitor negative control (Ctr) or miR-101 inhibitor (inhibitor) was measured by using western blot. (G) The Luciferase activity was analyzed in 293T cells co-transfected with wild type (PKcs-WT) or mutant (PKcs-mut) 3'UTR of DNA-PKcs and Ctr or miR-101. **P<0.01, compared with adjacent tissues; ^##^P<0.01, compared with HPDE6C7 cells.

We previously found that miR-101 silences DNA-PKcs and sensitizes pancreatic cancer cells to gemcitabine 13. MiR-101 was demonstrated to be a tumor suppressor in various cancers, including PDAC 26-29. Thus, we investigated whether DNA-PKcs serves as a direct target of miR-101 to regulate the malignant behaviors of PDAC cells. As shown in Fig. 3A, there was a potential binding site between miR-101 and 3′-UTR of DNA-PKcs. The expression of miR-101 was down-regulated in PDAC tissues (Fig. 3B) and negatively correlated with that of DNA-PKcs (Fig. 3C). The miR-101 expression was also down-regulated in PDAC cell lines compared to HPDE6C7 cells (Fig. 3D). Western blot result showed that transfection of miR-101 mimic significantly inhibited DNA-PKcs expression in PANC-1 and MIA PaCa-2 cells, which showed low expression level of miR-101 (Fig. 3E). Transfection of miR-101 inhibitor obviously up-regulated DNA-PKcs expression in HPDE6C7 cells, which showed high expression level of miR-101 (Fig. 3F). Dual-luciferase gene reporter assay showed that the relative luciferase was markedly decreased in the cells co-transfected of miR-101 with wild type (WT) 3'UTR of DNA-PKcs, whereas no apparent change of the relative luciferase was observed in the cells co-transfected of miR-101 with mutation of the binding sequence (Fig. 3G). These data suggest that DNA-PKcs is a direct target of miR-101 in PDAC cells.

**Figure 4:**
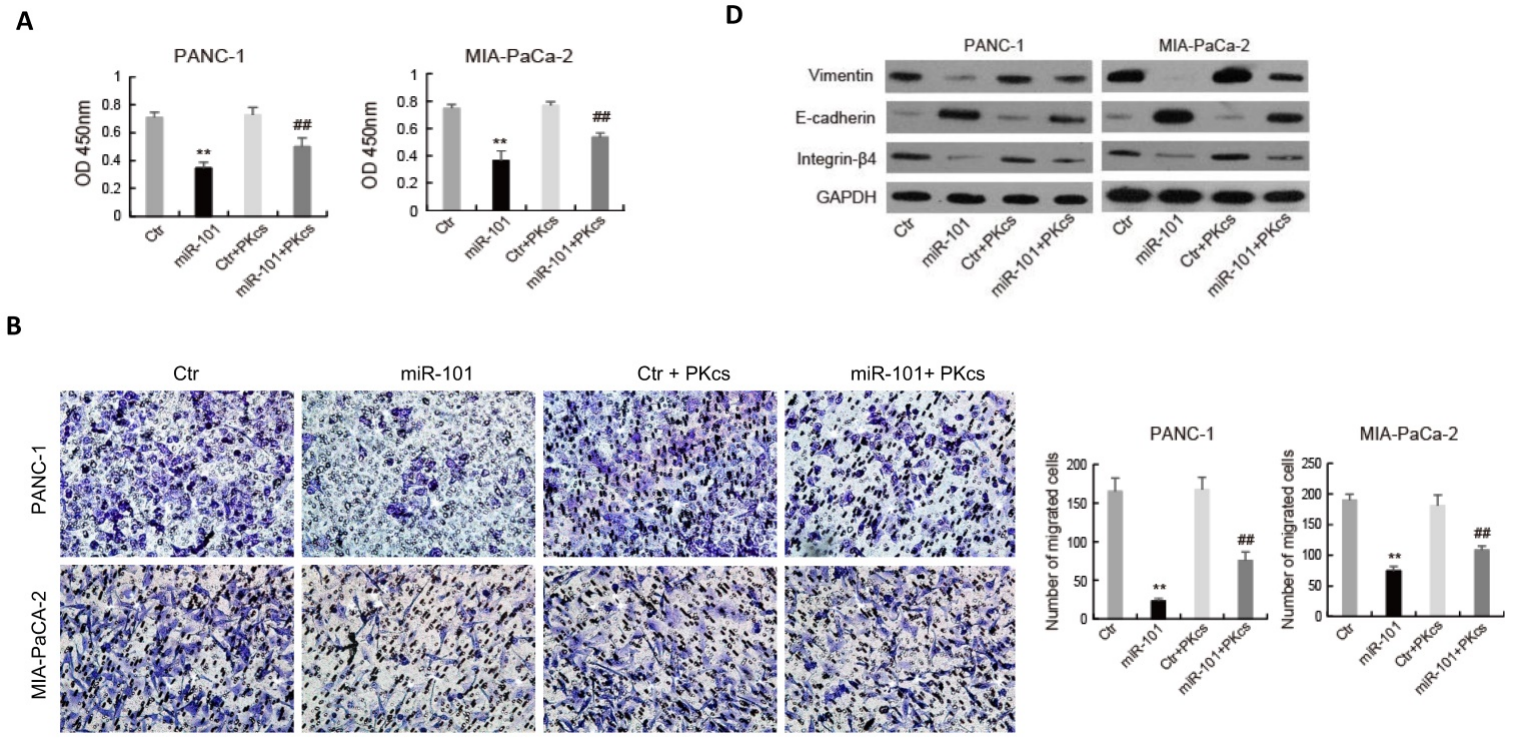

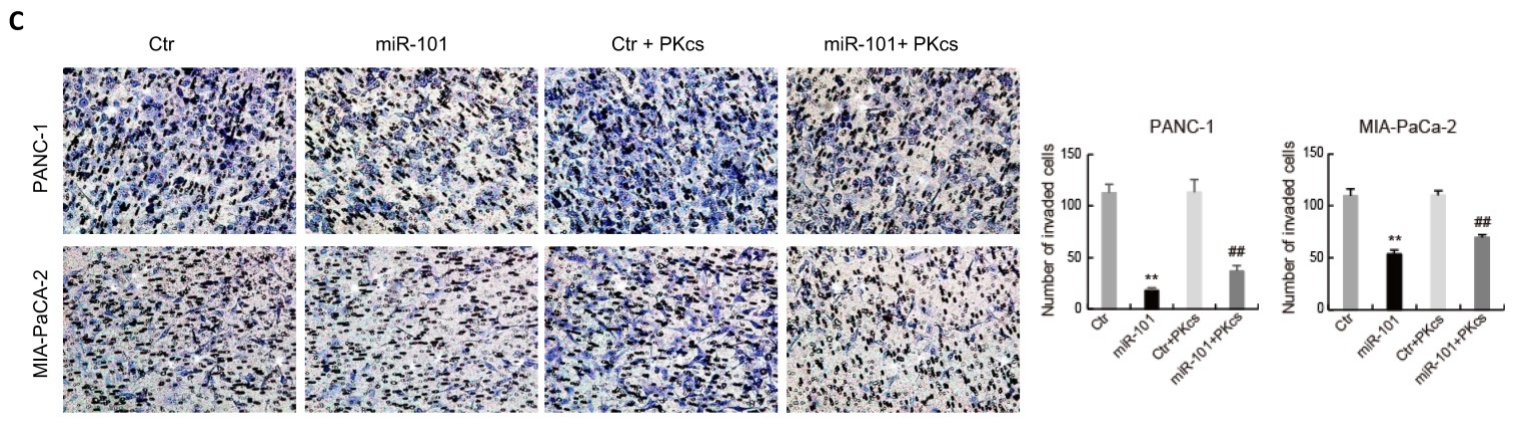
** miR-101 overexpression inhibits malignant behaviors of PDAC cells by regulating DNA-PKcs expression.** PANC-1 and MIA PaCa-2 cells transfected with miR-101 or rescued with DNA-PKcs (miR-101+PKcs) as indicated were subjected to CCK-8 assay (A), cell migration assay (B), cell invasion assay (C) and EMT markers examination (D).**P<0.01 compared with Ctr; ^#^P < 0.05, ^##^P < 0.01 compared with miR-101.

The effects of miR-101 on the biological behaviors of PDAC cells were further evaluated. As shown in Fig. 4, transfection of miR-101 mimic significantly inhibits proliferation (Fig. 4A), migration (Fig. 4B), invasion (Fig. 4C) and EMT (Fig. 4D) in PANC-1 and MIA PaCa-2 cells. Moreover, DNA-PKcs overexpression in the miR-101‑up-regulated PANC-1 and MIA PaCa-2 cells resulted in the accelerations of cell proliferation (Fig. 4A), migration (Fig. 4B), invasion (Fig. 4C) and EMT (Fig. 4D). These data suggest that DNA-PKcs serves as a direct target of miR-101 to regulate the malignant behaviors of PDAC cells.

### NEAT1 serves as a sponge of miR-101 to negatively regulate its expression

**Figure 5:**
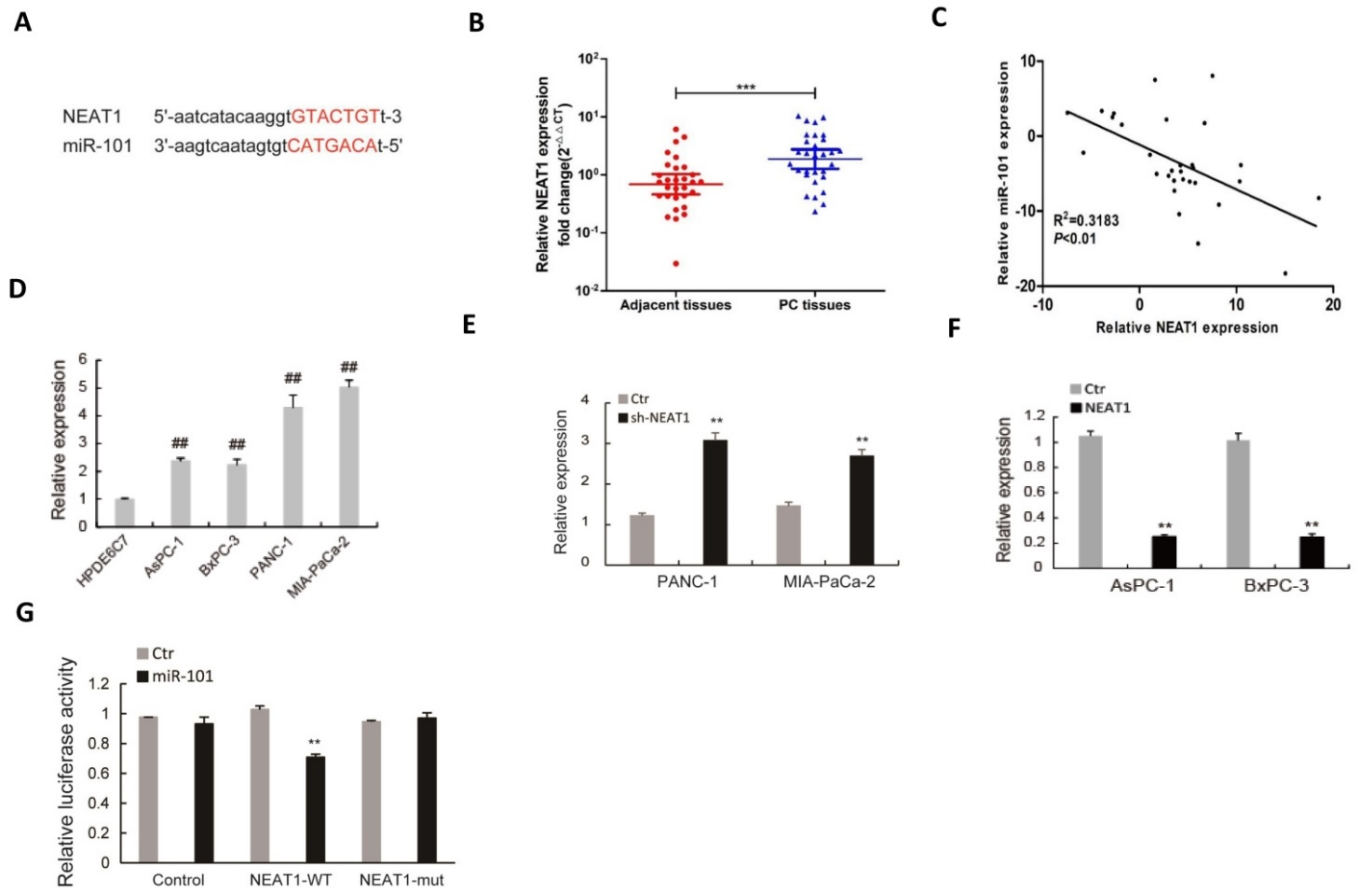
** NEAT1 serves as a sponge of miR-101 to negatively regulate its expression.** (A) The binding condition between NEAT1 and miR-101 were predicted, and the putative binding sites was indicated by red bases. (B) The level of NEAT1 was determined by qRT-PCR in 30 paired PDAC tissues. (C) The correlation analysis of miR-101 and NEAT1 expression. (D) The expression level of NEAT1 was determined by qPCR in 4 PDAC cell lines (AsPC-1, BxPC-3, MIA PaCa-2 and PANC-1) and normal pancreatic cells (HPDE6C7). (E) The expression of miR-101 in PANC-1 and MIA PaCa-2 cells transfected with NEAT1 shRNA (sh-NEAT1) or control shRNA (Ctr) was measured using RT-qPCR. (F) The expression of miR-101 in AsPC-1 and BxPC-3 cells transfected with NEAT1 overexpressed plasmid (NEAT1) or control plasmid (Ctr) was measured using RT-qPCR. (G) The Luciferase activity was analyzed in 293T cells co-transfected with wild type (NEAT1-WT) or mutant (NEAT1-mut) binding sites of NEAT1 and miRNA negative control (Ctr), miR-101 mimic (miR-101). **P<0.01compared with Ctr; ^##^P<0.01 compared with HPDE6C7.

The regulatory mechanism of miR-101 expression was further investigated. As shown in Fig 5A, there is a potential binding site of NEAT1 to miR-101. The expression of NEAT1 was up-regulated in PDAC tissues (Fig. 5B) and negatively correlated with that of miR-101 (Fig. 5C). The NEAT1 expression was also up-regulated in PDAC cell lines compared to HPDE6C7 cells (Fig. 5D). RT-qPCR showed that down-regulation of NEAT1 by shRNA promoted miR-101 expression in PANC-1 and MIA PaCa-2 cells, which showed high expression level of NEAT1 (Fig. 5E). NEAT1 overexpression obviously inhibited miR-101 expression in AsPC-1 and BxPC-3 cells, which showed low expression level of NEAT1 (Fig. 5F). Dual-luciferase gene reporter assay revealed that the relative luciferase was significantly decreased in HEK293 cells co-transfected of miR-101 with NEAT1 that contained wild type miR-101 binding site (WT-NEAT1), whereas no apparent change of the relative luciferase was observed in the cells co-transfected of miR-101 with NEAT1 that contained mutant miR-101 binding site (MUT-NEAT1) (Fig. 5G). These data suggest that NEAT1 interacted with miR-101 and negatively regulated its expression.

### NEAT1 regulates the malignant behaviors of PDAC cells through miR-101/DNA-PKcs

**Figure 6:**
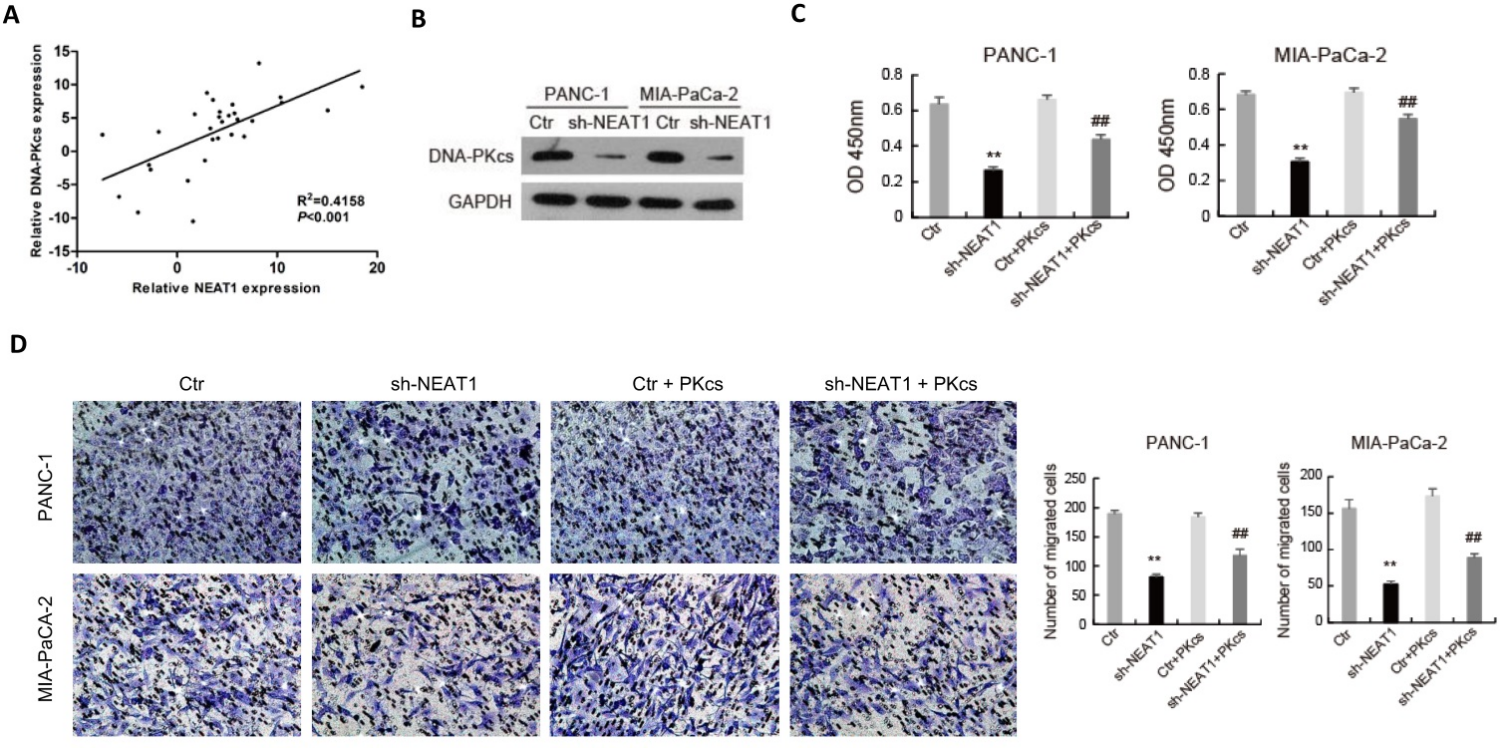

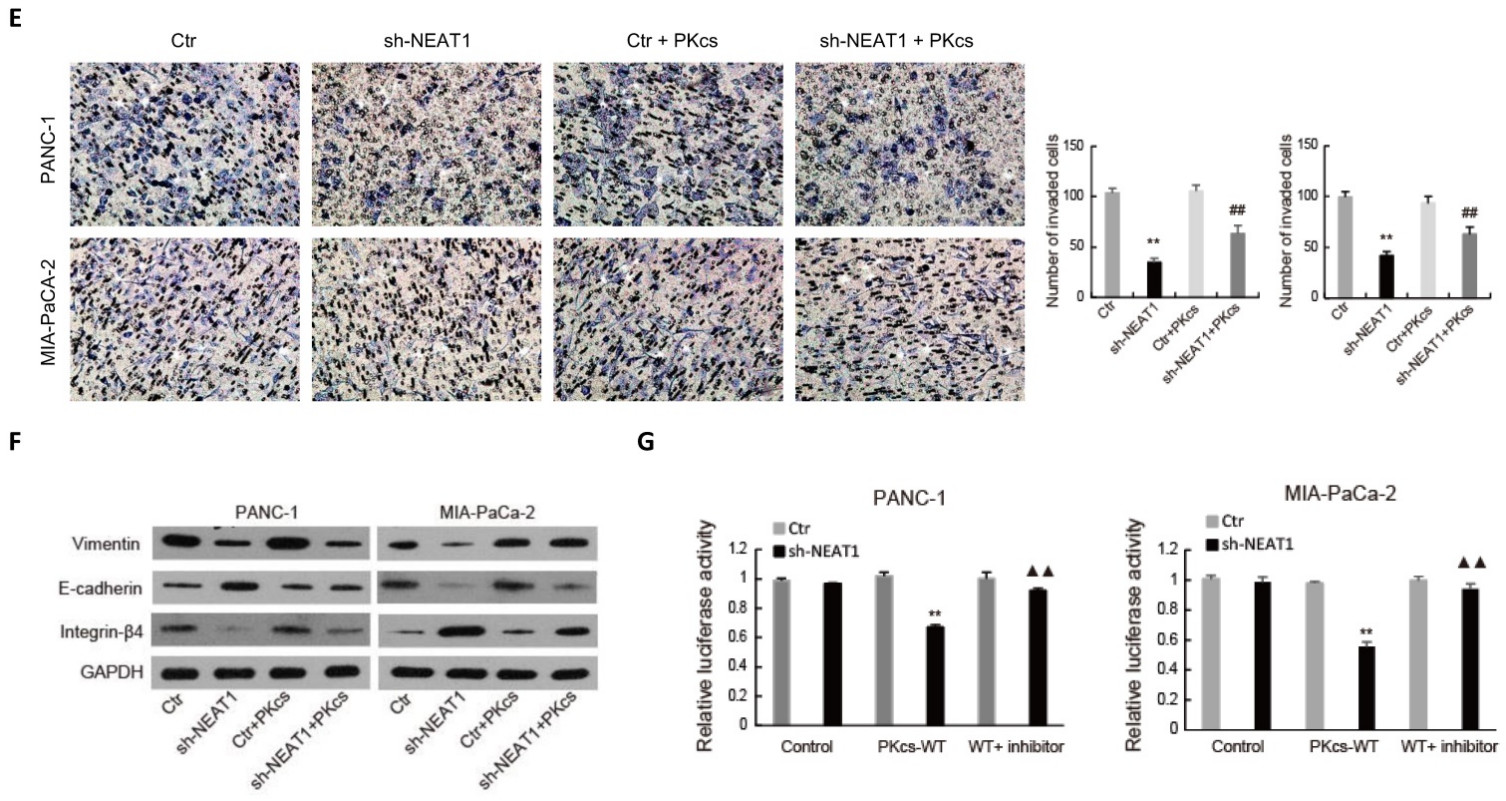
** NEAT1 regulates the malignant behaviors of PDAC cells through miR-101/ DNA-PKcs.** (A) The correlation analysis of NEAT1 and DNA-PKcs expression. (B) The expression of DNA-PKcs in PANC-1 and MIA PaCa-2 cells transfected with NEAT1 shRNA (sh-NEAT1) or control shRNA (Ctr) was measured using western blot. (C-F) PANC-1 and MIA PaCa-2 cells transfected with sh-NEAT1 or rescued with DNA-PKcs (sh-NEAT1+PKcs) as indicated were subjected to CCK-8 assay (C), cell migration assay (D), cell invasion assay (E) and EMT markers examination (F). (G) The Luciferase activity was analyzed in PANC-1 and MIA PaCa-2 cells co-transfected with PKcs-WT and sh-NEAT1 or rescued with miR-101 inhibitor (WT+inhibitor). **P<0.01compared with Ctr; ^##^P < 0.01 compared with sh-NEAT1; ^▲▲^P < 0.01 compared with sh-NEAT1+ PKcs-WT.

As shown in Fig. 6A, the expression level of NEAT1 was positively correlated with that of DNA-PKcs in PDAC tissues. To further elucidate the effects of NEAT1 on the malignant behaviors of PDAC cells, endogenous NEAT1 expression was inhibited by shRNA in PANC-1 and MIA PaCa-2 cells, which showed high expression level of NEAT1. As shown in Fig. 6B, DNA-PKcs was found to be significantly decreased in PANC-1 and MIA PaCa-2 cells after the transfection of NEAT1 shRNA. To determine whether NEAT1 and DNA-PKcs regulate the malignant behaviors of PDAC cells through the same functional pathway, DNA-PKcs was overexpressed in NEAT1‑silenced PANC-1 and MIA PaCa-2 cells. As shown in Fig. 6C-6F, DNA-PKcs overexpression in the NEAT1‑silenced PANC-1 and MIA PaCa-2 cells resulted in the accelerations of cell proliferation (Fig. 6C), migration (Fig. 6D), invasion (Fig. 6E) and EMT (Fig. 6F). These data suggested that NEAT1 likely exerts its effects on the malignant behaviors of PDAC cells through up-regulation of DNA-PKcs.

The regulatory relationship among NEAT1, miR-101 and DNA-PKcs in PDAC cells was further investigated. Dual-luciferase gene reporter assay showed that the relative luciferase activity of WT- DNA-PKcs was obviously reduced by NEAT1 knockdown, while the inhibitory effect of NEAT1 knockdown was partially attenuated by miR-101 inhibition in PANC-1 and MIA PaCa-2 cells (Fig. 6G). These data suggest that NEAT1 regulates the malignant behaviors of PDAC cells through miR-101/ DNA-PKcs.

## Discussion

Although emerging evidence demonstrates that DNA-PKcs overexpression plays an important role in progression and metastasis in multiple cancers 6, 11, 31, 32, the effects and mechanism of its overexpression on the malignant behaviors of PDAC cells are still unclear. In the present study, we showed that DNA-PKcs expression was significantly elevated in human PDAC tissues and cells. DNA-PKcs overexpression not only was correlated with TNM stage and lymph node metastasis, but also enhanced the abilities of PDAC cell proliferation, migration and invasion. To the best of our knowledge, the present study firstly reported that DNA-PKcs overexpression play a critical role in the malignant behaviors of PDAC cells. A previous study has revealed that depletion of DNA-PKcs impairs melanoma cell migration and invasion through regulation of secreted proteins involved in migration and invasion, such as TIMP-2, α-2M, MMP-8, and MMP-14 32. The study by Goodwin *et al.* showed that DNA-PKcs promotes prostates cancer cell migration and invasion by functioning as a selective modulator of transcriptional networks 6. In addition, DNA-PKcs inhibition in renal cell carcinoma cells suppresses cell proliferation by inhibiting AKT Ser-473 phosphorylation and HIF-2α expression 31. These findings suggest that the mechanism by which DNA-PKcs regulates cancer cell proliferation, migration and invasion may depend on the cell type. The possible mechanisms involved in the inhibition of PDAC cell proliferation, migration and invasion induced by DNA-PKcs silencing need to be explored in the further.

Further study of the underlying mechanisms involved in DNA-PKcs overexpression, suggested that miR-101 was involved. We focused our investigations on miR-101 as we previously found that miR-101 targeted 3′UTR of DNA-PKcs mRNA 13 and miR-101 served as a tumor suppressor in PC 33-35. Consistent with previous studies, we found that miR-101 was decreased in PDAC tissues and cells. Our results showed that introduction of miR-101 in PDAC cells down-regulated DNA-PKcs expression. Moreover, miR-101 overexpression in PDAC cells markedly suppressed cell proliferation, migration and invasion, which were rescued partially by DNA-PKcs overexpression. These data suggest that miR-101 down-regulation might be at least one key reason for DNA-PKcs overexpression in PDAC and DNA-PKcs serves as a direct target of miR-101 to regulate the malignant behaviors of PDAC cells. We previously demonstrated that miR-101 silences DNA-PKcs and sensitizes PDAC cells to gemcitabine 13. All these findings suggested that miR-101/ DNA-PKcs may play an important role in the progression of PDAC.

It is reported that NEAT1 contributes development and progression of cancer by sponging miRNAs 18, 20-22. NEAT1 was demonstrated to interact with miR-101 and inhibit its expression through a ceRNA mechanism in multiple cancer cells 25-29. Importantly, NEAT1 was shown to be up-regulated in PDAC and important for PDAC cell migration and invasion 23. We then investigated whether increased PDAC cell proliferation, migration and invasion that induced by miR-101-mediated DNA-PKcs up-regulation were due to NEAT1. In the present study, NEAT1 was found to be up-regulated in PDAC tissues and cells. Our results indicated that NEAT1 serves as a sponge of miR-101 to negatively regulate its expression in PDAC cells. DNA-PKcs overexpression could partially reversed NEAT1 knockdown-induced decrease of PDAC cell proliferation, migration and invasion. These data indicated that increased NEAT1 in PDAC cells up-regulated DNA-PKcs expression by sponging miR-101, leading to increase of PDAC cell proliferation, migration and invasion. Recently, NEAT1 was shown to promote PC cell proliferation and metastasis through stabilizing ELF3 mRNA 22. A previous study demonstrated that NEAT1 formed a feedback loop with RELA and miR-302a-3p to promote PDAC cell migration and proliferation 23. The study by Huang *et al.* revealed that NEAT1 facilitates PC cell proliferation, migration and invasion at least partly through negative modulation of miR-506-3p 36. NEAT1 was also demonstrated promote PC cell growth, invasion and migration though miR-335-5p/c-met axis 37. Our and these findings suggest that NEAT1 serves as a sponge of different miRNAs to facilitate PC cell proliferation, migration and invasion. Further researches are needed to investigate whether there is a crosstalk between these targeted miRNAs of NEAT1 and how these different downstreams of NEAT1 work together to promote PDAC cell proliferation, migration and invasion.

In conclusion, the present study demonstrated that NEAT1/miR-101-dependent up-regulation of DNA-PKcs promotes the malignant behaviors of PDAC cells and provided a new therapeutic strategy to treat PDAC. However, further *in vivo* studies are needed to confirm the promotive effect of NEAT1/miR-101/ DNA-PKcs axis on the malignant behaviors of PDAC cells.

## Figures and Tables

**Table 1:** Correlation between DNA-PKcs expression and clinicopathologics of PC patients^a^.

Characteristics	N of cases	DNA-PKcs level		*P*-value
		Low	High	
Total cases	30	15	15	1.000
Gender				
Male	19	9	10	
Female	11	6	5	
Age				1.000
<60	9	5	4	
≥60	21	10	11	
TNM stage(AJCC)^b^				0.013^✱^
I	16	12	4	
II	12	3	9	
III	2	0	2	
T stage				0.545
T1	5	4	1	
T2	19	9	10	
T3	5	2	3	
T4	1	0	1	
Lymph node metastasis				0.035^✱^
Negative	22	14	8	
Positive	8	1	7	

Abbreviations: N of cases = number of cases; TNM = tumor node metastasis; T stage = tumor stage. ^a^ Fisher's exact test or Chi-Square test, if appropriate, ^✱^*P*<0.05. ^b^ American Joint Committee on Cancer (AJCC), patients were staged in accordance with the 8^th^ Edition of the AJCC Cancers' TNM Classification.

## Abbreviations

DNA-PKcs: DNA-dependent protein kinase catalytic subunit
PDAC: pancreatic ductal adenocarcinoma
NEAT1: nuclear paraspeckle assembly transcript 1
OS: overall survival
ceRNAs: competing endogenous RNA
PC: pancreatic cancer
PIKK: phosphatidylinositol-3 kinase-like protein kinase
